# Prediction of cardiovascular events and all-cause mortality using race and race-free estimated glomerular filtration rate in African Americans: the Jackson Heart Study

**DOI:** 10.3389/fmed.2024.1432965

**Published:** 2024-10-31

**Authors:** Haiping Wang, Jiahui Cai, Hao Fan, Clarissa J. Diamantidis, Bessie A. Young, Aurelian Bidulescu

**Affiliations:** ^1^Key Laboratory of Biotherapy and Regenerative Medicine of Gansu Province, First Hospital of Lanzhou University, Lanzhou, China; ^2^Department of Epidemiology and Biostatistics, Indiana University School of Public Health, Bloomington, IN, United States; ^3^Department of Medicine, Duke University School of Medicine, Durham, NC, United States; ^4^Division of Nephrology, Department of Medicine, University of Washington, Seattle, WA, United States; ^5^Justice, Equity, Diversity, and Inclusion Center for Transformational Research, Office of Healthcare Equity, University of Washington, Seattle, WA, United States

**Keywords:** estimate glomerular filtration rate, creatinine, cystatin C, cardiovascular disease, all-cause mortality, African Americans

## Abstract

**Background:**

New Chronic Kidney Disease Epidemiology Collaboration (CKD-EPI) equations without a race adjustment were developed to estimate the glomerular filtration rate (eGFR). We aimed to compare the performance of five CKD-EPI eGFR equations, with or without race, in predicting cardiovascular disease (CVD) events and all-cause mortality in Black Americans from the Jackson Heart Study.

**Methods:**

JHS is an ongoing population-based prospective cohort study of African Americans in the Jackson, Mississippi, metropolitan area. Five CKD-EPI equations were used to estimate GFR at baseline using serum creatinine (Cr) or cystatin C (cys), including 2009 eGFRcr(ASR [age, sex, race]), 2021 eGFRcr(AS [age and sex]), 2012 eGFRcr-cys(ASR), 2021 eGFRcr-cys(AS), 2012 eGFRcys(AS). Endpoints were incident CVD events and all-cause mortality. Cox proportional hazards regression was used to assess the associations between different eGFRs and outcomes adjusting for atherosclerotic risk factors. Harrell’s C-statistics and Net Reclassification Index (NRI) were used to assess the predictive utility.

**Results:**

Among 5,129 participants (average age 54.8 ± 12.8 yrs), 1898 were male (37.0%). eGFRcr(AS) provided lower estimates and resulting in a greater proportion of participants categorized as CKD than eGFRcr(ASR), eGFRcr-cys(ASR), eGFRcr-cys(AS) and eGFRcys(AS). A median follow-up of 13.7 and 14.3 years revealed 411 (9.3%) CVD incidents and 1,207 (23.5%) deaths. Lower eGFRs were associated with CVD incidents and all-cause mortality. eGFRcr-cys(ASR), eGFRcr-cys(AS) and eGFRcys(AS) were strongly associated with incident CVD events and all-cause mortality than eGFRcr(ASR) and eGFRcr(AS). A significant discrimination improvement was found in C-statistics for predicting incident CVD events and all-cause mortality after adding each eGFR measure to the basic model including atherosclerotic risk factors. Across a 7.5% 10-year risk threshold, eGFRcys(AS) improved net classification of all-cause mortality (NRI: 2.19, 95%CI: 0.08, 4.65%).

**Conclusion:**

eGFR based on creatinine omit race has the lowest mean and detects more CKD patients in Black population. The eGFRs incorporating cystatin C strengthens the association between the eGFR and the risks of incident CVD and all-cause mortality. Cystatin C-based eGFR equations might be more appropriate for predicting CVD and mortality among Black population.

## Introduction

Chronic kidney disease (CKD) has been recognized as a major public health problem that is an important risk factor for cardiovascular disease (CVD) and all-cause mortality (1). CKD affects more than 10% of the general population around the world, accounting for more than 800 million (2). In the United States, 15% of adults were estimated to have CKD, and CKD is more common in non-Hispanic Black adults than in non-Hispanic White or Asian adults (3).

Glomerular filtration rate (GFR) is generally accepted as the best overall index of kidney function in disease and health and is recommended as a principal measure to define CKD and categorize the damage stage of CKD in guidelines (4). The equations commonly used for eGFR are the serum creatinine (cr) based Modification of Diet in Renal Disease (MDRD) study equation (5) and the Chronic Kidney Disease Epidemiology Collaboration (2009 CKD-EPI eGFRcr) equation (6), the serum cystatin C (cys) based CKD-EPI (2012 CKD-EPI eGFRcys) and the combination of creatinine and cystatin C based CKD-EPI (2012 CKD-EPI eGFRcr-cys) (7). 2009 CKD-EPI eGFRcr and 2012 CKD-EPI eGFRcr-cys incorporate race in calculation. However, recognizing the limitations of race-based medicine and an evolved consideration of race as a social, not biological construct, new non-race-based equations were derived, including 2021 CKD-EPI eGFRcr and 2021 CKD-EPI eGFRcr-cys (8). The National Kidney Foundation (NKF) and the American Society of Nephrology (ASN) convened a Task Force to review the inclusion of race in eGFR and recommended immediate implementation of CKD-EPI new race-free eGFRcr or eGFRcr-cys equations for kidney function estimation (9).

The Jackson Heart Study (JHS) is a population-based cohort study of African Americans, with a high prevalence of diabetes, CKD, and cardiovascular diseases (10). Studies have reported that lower eGFR is significantly associated with a higher risk of all-cause mortality and cardiovascular diseases independently of other traditional risk factors (11–16), and lower eGFR has been demonstrated as a significant risk factor for CVD and mortality in JHS (17). Recently, a new Predicting Risk of Cardiovascular Disease Events (PREVENT) equation was released by the American Heart Association (AHA) including eGFR as well as traditional risk factors, the eGFR was calculated using 2021 CKD-EPI eGFRcr (18). Accordingly, we aimed to compare the performance of different CKD-EPI eGFR equations with and without race in predicting CVD events and all-cause mortality in JHS participants.

## Methods

### Study sample

**Figure 1:**
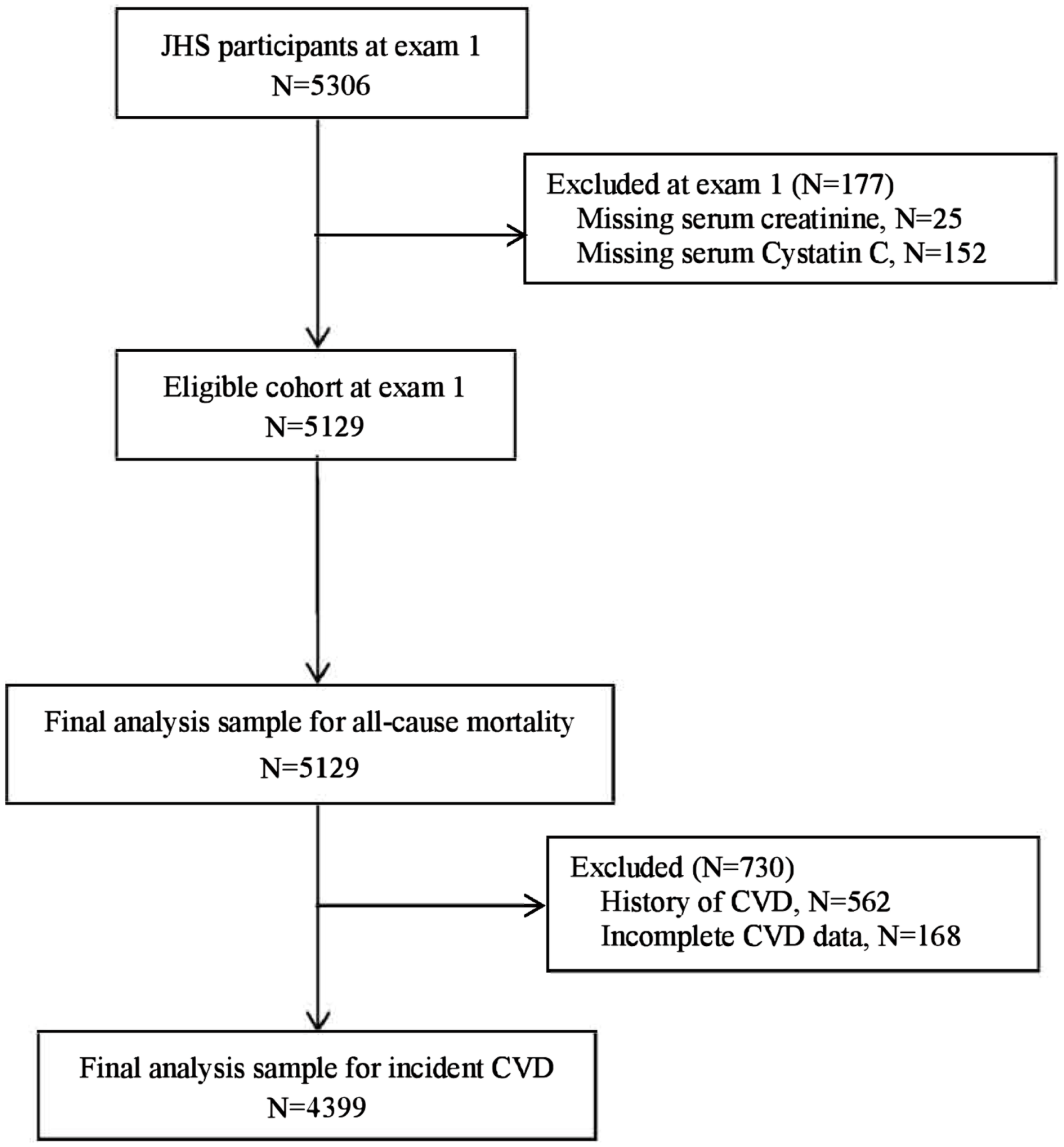
Flow diagram.

The JHS is an ongoing community-based, prospective cohort study intended to evaluate CVD risk among self-reported African Americans. The details of the study have been published previously (19, 20). The original JHS cohort enrolled participants from September 2000 to March 2004 (exam 1) and comprised 5,306 participants with 20–95 years. For the current analysis, participants with complete serum creatinine and cystatin C at exam 1 were included, *n* = 5,129 (excluding 25 participants with missing serum creatinine, and 152 participants missing cystatin C). For incident CVD, an additional 730 participants were excluded (168 participants had incomplete CHD and stroke information and 562 participants had CHD or stroke at baseline). Finally, 4,399 participants and 5,129 participants were included in the incident CVD and all-cause mortality analyses (Figure 1), respectively. The study was approved by the institutional review board of the participating institutions (University of Mississippi Medical Center, Jackson State University, and Tugaloo College). All of the participants provided informed consent.

### eGFR

Serum creatinine (mg/dl) and cystatin C (mg/L) were collected at exam 1. Serum creatinine was assessed using an enzymatic method on a Vitros 950 (21) and calibrated to the isotope-dilution mass spectrometry (IDMS) traceable method (22). Serum cystatin C was measured by a particle-enhanced immunonephelometric assay. Five CKD-EPI equations were considered in this study (Supplementary Table S1), for calculating eGFRs based on serum creatinine or cystatin C with and without race (8), including 2009 CKD-EPI eGFRcr(ASR [age, sex, and race]), 2021 CKD-EPI eGFRcr(AS [age and sex]), 2012 CKD-EPI eGFRcr-cys(ASR), 2021 CKD-EPI eGFRcr-cys(AS), 2012 CKD-EPI eGFRcys(AS).

### Outcomes

The outcomes were incident CVD and all-cause mortality. Incident CVD was defined as the first occurrence of coronary heart disease (CHD) or stroke among participants without CVD at baseline. Annual follow-up interviews and medical record reviews were used to adjudicate CHD, stroke, and all-cause mortality from 2000, whereas the adjudication of heart failure (HF) which was excluded from our measure of incident CVD began in 2005. The detailed adjudication description has been published previously (23). At the time of analyses for this study, incident CVD events were adjudicated through December 31, 2016, and all-cause mortality was assessed through December 31, 2018.

### Covariate assessments

All basic characteristics and clinical covariates were measured at baseline, including the following: age (years); sex; body mass index (BMI) in kg/m^2^; current smoker; alcohol; income; education; systolic blood pressure (SBP); diastolic blood pressure (DBP); hypertension; diabetes; history of CVD (coronary heart disease, stroke or both); self-reported history of CKD; medication status of hypertension, diabetes and statin; fasting plasma glucose (mg/dl); total cholesterol (mg/dl); high-density lipoproteins (HDL) cholesterol (mg/dl); low-density lipoproteins (LDL) cholesterol (mg/dl); triglycerides (mg/dl); high-sensitivity C-reactive protein (hsCRP, mg/dl). Sitting blood pressure was measured twice at 1-min intervals after 5-min resting and the average of two measurements was used for analysis. Hypertension was defined as blood pressure ≥ 140/90 mmHg or the use of antihypertension medications. Diabetes was defined as fasting glucose ≥126 mg/dl or hemoglobin A1c ≥ 6.5% or the use of diabetic medications. CKD was defined as the eGFR less than 60 ml/min/1.73 m^2^ (4).

### Statistical analysis

Participants were classified according to 2009 eGFRcr(ASR) categories (≥90, 60–90, <60 ml/min/1.73m^2^). Continuous variables were expressed as the mean (standard deviation, SD) or median (interquartile range, IQR), and categorical variables were expressed as numbers (percentages). Tests for trends across categories were conducted using linear regression, the Jonckheere-Terpstra test, or the Cochran-Armitage trend test, as appropriate.

A kernel density plot was conducted to display the distribution of different eGFRs. Event-free survival curves for each outcome were plotted using the Kaplan–Meier method. To explore the relationship between each eGFR and outcome, restricted cubic splines, with 8 knots at eGFR of 20, 30, 45, 60, 75, 90, 105, and 115 ml/min/1.73m^2^, were conducted using Cox proportional hazard regression adjusted for atherosclerotic risk factors (age, sex, current smoking, SBP, DBP, diabetes, total cholesterol, HDL cholesterol, antihypertensive medication, and statin medication) for incident CVD, and additionally CVD history for all-cause mortality. Atherosclerotic risk factors were from the American Heart Association/American College of Cardiology (AHA/ACC) guidelines (24). An eGFR of 90 ml/min/1.73m^2^ was considered the reference. Ethnicity was not included as our participants are Black.

Cox regression was used to assess hazard ratios (HRs) and 95% confidence intervals (CIs) for incident CVD events and all-cause mortality, and different eGFRcr categories as independent variables. Two models were generated using the following stages: Model 1 adjusted for age and sex; Model 2 adjusted for atherosclerotic risk factors (age, sex, current smoking, SBP, DBP, diabetes, total and HDL cholesterol, antihypertensive, and statin medication) for incident CVD, and additionally CVD history for all-cause mortality. Harrell’s C-statistics was calculated to compare the predictive value after the addition of eGFRcr(ASR), eGFRcr(AS), eGFRcr-cys(ASR), eGFRcr-cys(AS), eGFRcys(AS) to base multivariable model which included atherosclerotic risk factors for incident CVD, and additionally CVD history for all-cause mortality. Net Reclassification Index (NRI) was calculated for the reclassification of participants from “low” to “intermediate” risk, across the threshold 7.5% 10-year risk of CVD (24). The 95% CIs were obtained from 1,000 bootstrap replicates. Analyses were performed using SAS 9.4 (SAS Institute) and the nricens package in R studio (version 4.3.2) for NRI. A 2-sided *p*-value of 0.05 was considered statistically significant.

## Results

### Demographics of characteristics

A total of 5,129 JHS participants were included in the current study presented in Table 1. The average age was 54.8 ± 12.8 years and 1898 (37.0%) were male. The details about baseline and clinical characteristics of participants were compared according to the 2009 eGFRcr(ASR) categories. Participants with lower eGFR had higher age, waist, SBP, fasting plasma glucose, HbA1c, total cholesterol, LDL, triglycerides and lower DBP (P for trend over eGFR categories <0.01). Non-current smokers, lower alcohol intake and education attainment were more common in those participants with lower eGFR (P for trend <0.001). Lower eGFR participants were more likely to have hypertension, diabetes, CVD history, CKD history, and were more likely to take statins and medications for hypertension and diabetes (P for trend <0.001).

**Table 1:** Basic characteristics of our participants stratified by 2009 eGFRcr(ASR) categories.

Characteristics	Total	2009 eGFRcr(ASR) (ml/min/1.73m^2^)			
		≥90	60–90	<60	*p* for trend
N	5,129	3,116	1,694	319	
Demographics					
Age (years)	54.8 ± 12.8	50.3 ± 11.9	60.8 ± 10.8	67.2 ± 10.0	<0.001
Male	1,898 (37.0)	1,121 (36.0)	676 (39.9)	101 (31.7)	0.48
Current smoker	673 (13.2)	492 (15.9)	157 (9.3)	24 (7.6)	<0.001
Alcohol	2,352 (46.1)	1,621 (52.3)	657 (38.9)	74 (23.3)	<0.001
Education					
Less than high school	933 (18.3)	408 (13.1)	402 (23.8)	123 (38.9)	<0.001
High school/GED	1,032 (20.2)	620 (20.0)	346 (20.5)	66 (20.9)	
More than high school	3,145 (61.5)	2077 (66.9)	941 (55.7)	127 (40.2)	
Physical examination					
BMI (kg/m^2^)	31.8 ± 7.2	31.9 ± 7.6	31.4 ± 6.5	31.8 ± 6.8	0.07
Waist (cm)	100.8 ± 16.1	100.3 ± 16.9	100.9 ± 14.6	104.2 ± 15.3	<0.001
SBP (mmHg)	128 ± 17	125 ± 16	131 ± 17	134 ± 20	<0.001
DBP (mmHg)	76 ± 9	76 ± 9	76 ± 9	73 ± 10	0.002
Comorbidities					
Hypertension	2,902 (56.6)	1,473 (47.3)	1,146 (67.7)	283 (88.7)	<0.001
Diabetes	1,208 (23.6)	646 (20.8)	409 (24.2)	153 (48.0)	<0.001
History of CVD	562 (11.3)	236 (7.8)	217 (13.3)	109 (35.6)	<0.001
History of CKD	251 (4.9)	110 (3.5)	65 (3.9)	76 (24.2)	<0.001
Medications					
Hypertension	2,670 (52.5)	1,308 (42.4)	1,074 (63.9)	288 (91.4)	<0.001
Diabetes	810 (15.9)	419 (13.6)	265 (15.8)	126 (40.0)	<0.001
Statin	700 (13.8)	325 (10.5)	279 (16.6)	96 (30.6)	<0.001
Laboratory					
Fasting plasma glucose (mg/dl)	100.2 ± 33.4	99.5 ± 34.3	100.0 ± 30.3	109.1 ± 39.6	0.002
HbA1c (%)	6.0 ± 1.3	5.9 ± 1.3	6.0 ± 1.1	6.5 ± 1.5	<0.001
Total cholesterol (mg/dl)	199.4 ± 40.1	196.6 ± 39.1	202.9 ± 39.5	208.8 ± 50.1	<0.001
HDL (mg/dl)	51.7 ± 14.6	51.7 ± 14.2	51.9 ± 15.4	51.2 ± 15.3	0.998
LDL (mg/dl)	126.7 ± 36.6	124.8 ± 35.6	129.4 ± 36.7	132.2 ± 44.8	<0.001
Triglycerides (mg/dl)	90 (65, 127)	85 (60, 120)	95 (72, 132)	111 (80, 154)	<0.001
hsCRP (mg/dl)	0.26 (0.11, 0.57)	0.26 (0.11, 0.56)	0.25 (0.11, 0.53)	0.40 (0.17, 0.79)	0.06
Serum creatinine (mg/dl)	0.85 (0.75, 1.04)	0.75 (0.75, 0.94)	1.04 (0.85, 1.14)	1.43 (1.23, 1.81)	<0.001
Serum cystatin C (mg/L)	0.71 (0.62, 0.81)	0.66 (0.59, 0.73)	0.78 (0.69, 0.87)	1.18 (0.97, 1.48)	<0.001

Data are *n* (%) for categorical variables and mean (standard deviation) or median (25th, 75th percentile) for continuous variables. GED, general education development; BMI, body mass index; SBP, systolic blood pressure; DBP, diastolic blood pressure; CVD, cardiovascular disease; CKD, chronic kidney disease; HDL, high-density lipoproteins; LDL, low-density lipoproteins; hsCRP, high-sensitivity C-reactive protein; eGFR, estimated glomerular filtration rate.

**Figure 2:**
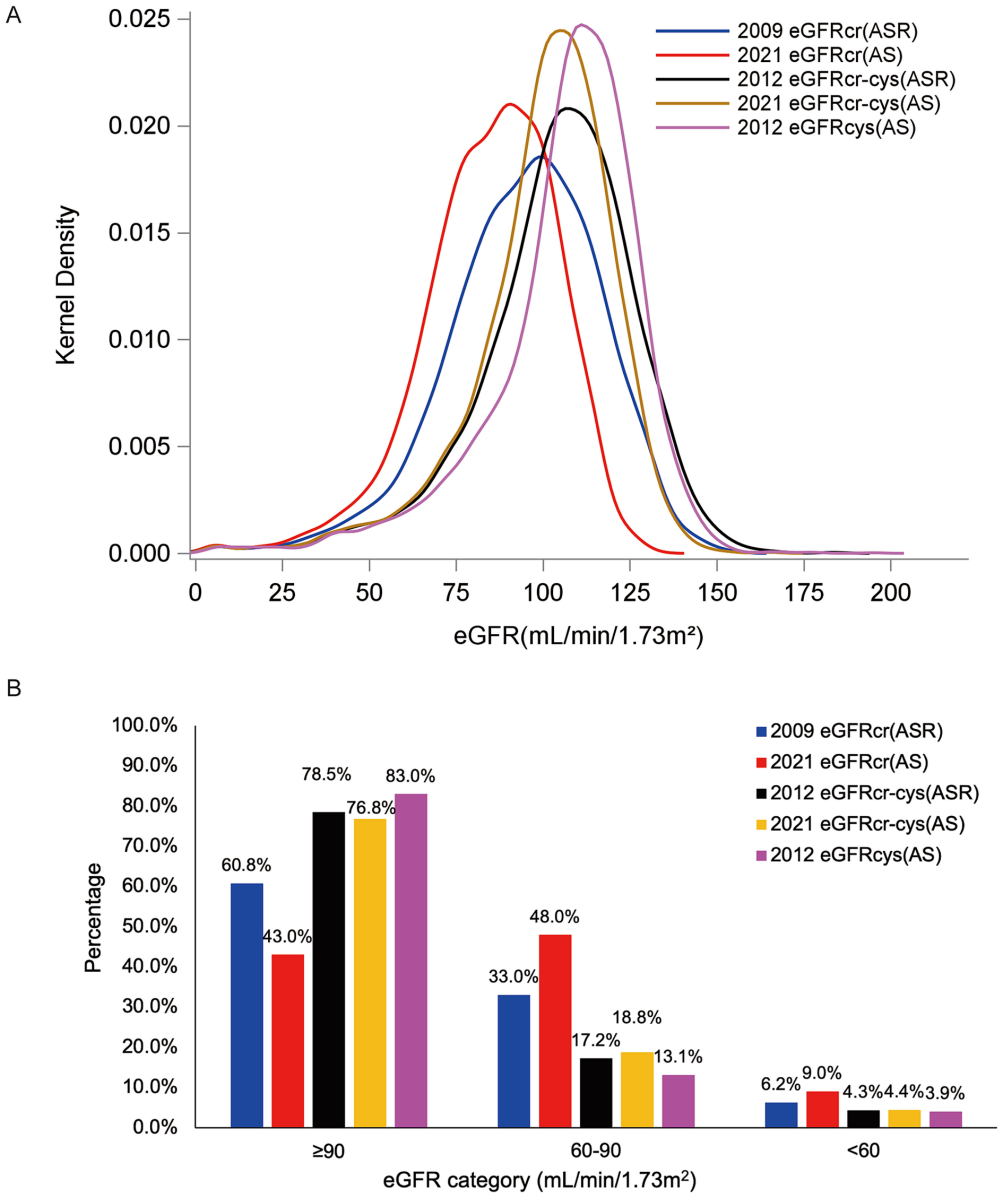
Distribution of eGFRs [**(A)** Kernel density of eGFR among our JHS participants. **(B)** Frequency of our participants in each eGFR category by eGFR estimating equations].

Among our participants, eGFRcr(AS) provided lower estimates than eGFRcr(ASR), eGFRcr-cys(ASR), eGFRcr-cys(AS) and eGFRcys(AS), and resulting in a greater proportion of participants categorized as CKD. Mean eGFR ± standard deviations were 94.4 ± 22.1, 84.9 ± 19.1, 104.1 ± 22.4, 100.4 ± 20.2, 106.6 ± 21.4 ml/min/1.73 m^2^ of eGFRcr(ASR), eGFRcr(AS), eGFRcr-cys(ASR), eGFRcr-cys(AS), eGFRcys(AS), respectively. The proportion of participants categorized as CKD was 6.2% for eGFRcr(ASR), 9.0% for eGFRcr(AS), 4.3% for eGFRcr-cys(ASR), 4.4% for eGFRcr-cys(AS) and 3.9% for eGFRcys(AS). The distribution of eGFR values was displayed in Figure 2A and the frequency of participants in each category was shown in Figure 2B.

### Associations between eGFR measures and incident CVD, all-cause mortality

**Figure 3:**
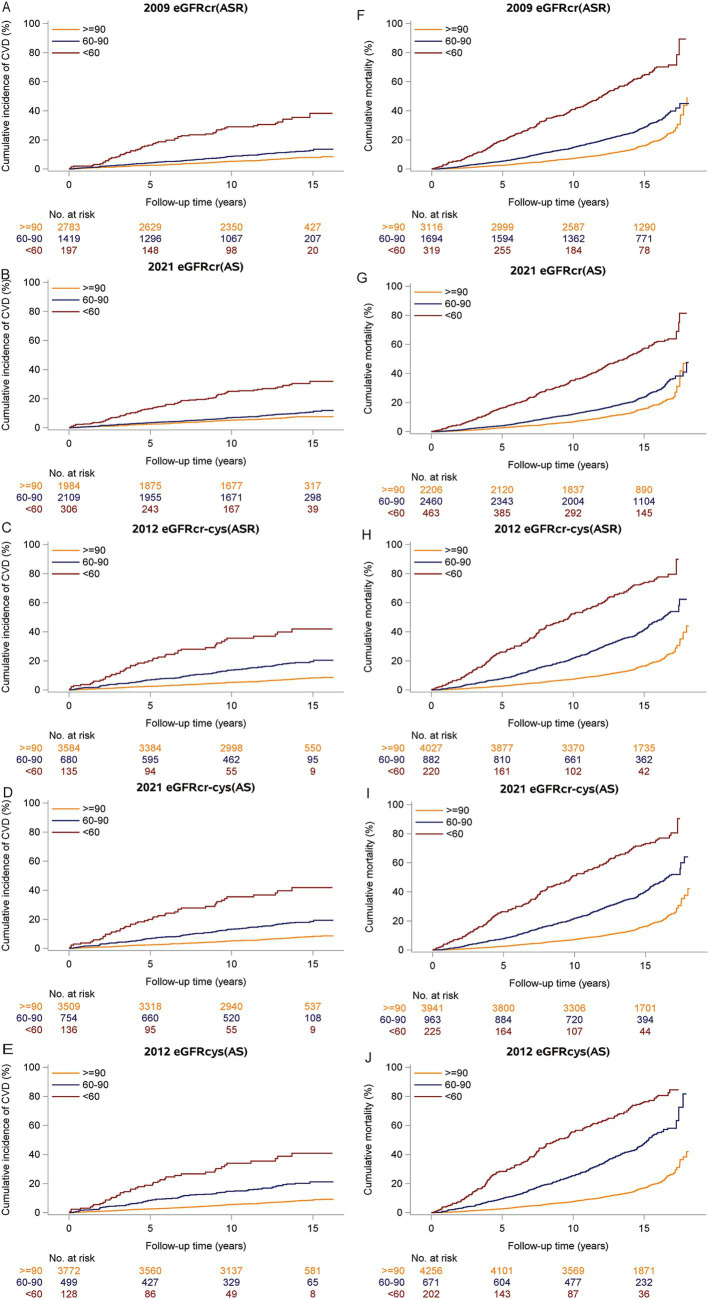
Cumulative incidence of CVD events and all-cause mortality in each eGFR category.

**Figure 4:**
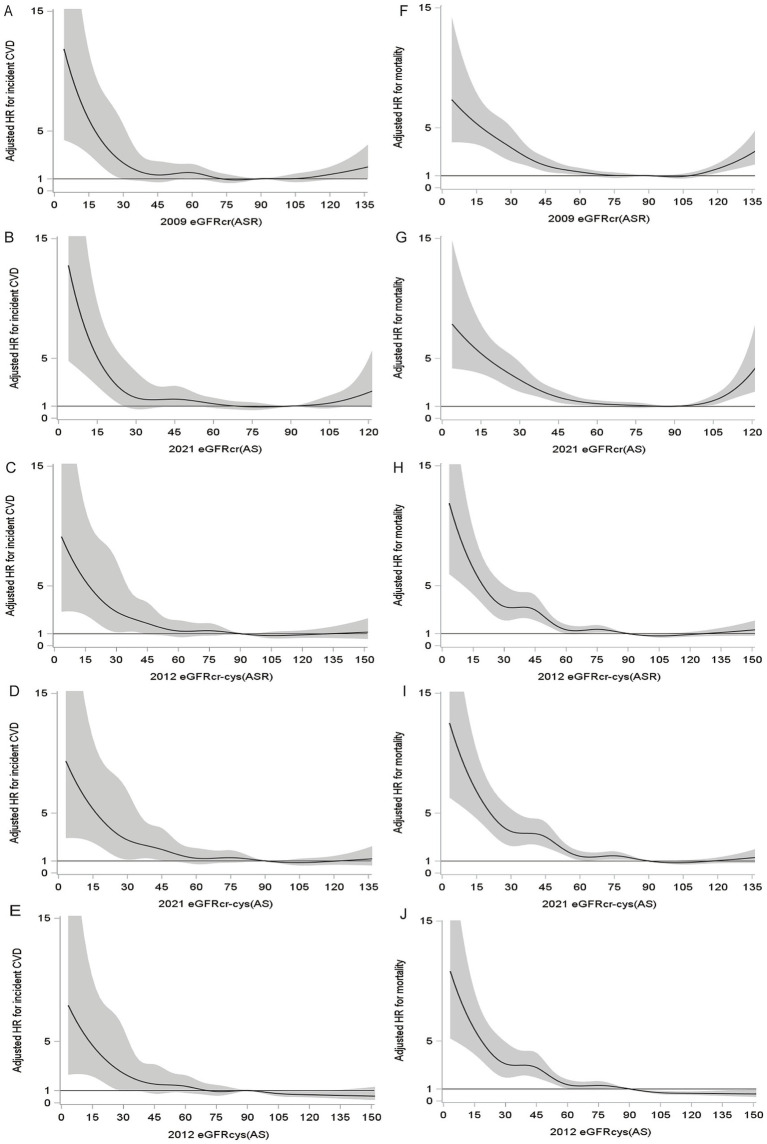
Fully adjusted splines of eGFR against adjusted hazard ratio (with 95% CI) for incident CVD and all-cause mortality. For incident CVD **(A–E)**, adjusted for atherosclerotic risk factors (age, sex, smoking, diabetes, SBP, DBP, hypertension medications, statin medications, total cholesterol, HDL). For all-cause mortality **(F–J)**, adjusted for atherosclerotic risk factors and CVD history.

During a median follow-up period of 13.7 years, 9.3% (411/4399) of participants had incident CVD. During a median follow-up for 14.3 years, 23.5% (1,207/5129) observed deaths were observed. The unadjusted cumulative incidence of incident CVD and all-cause mortality were shown in Figure 3, and both incidences gradually increased with decreasing eGFRs. The restricted cubic spline showed relationships between all eGFR measures and incident CVD (Figures 4A–E), which were negative and then relatively flat. The adjusted shape of the associations between eGFR measures and all-cause mortality displayed an initially negative association and then flat except for eGFRcr(ASR) and eGFRcr(AS), which increased mortality after 105 ml/min/1.73m^2^ (Figures 4H–J).

Adjusted HRs for the associations between eGFR measurements and incident CVD and all-cause mortality were consistent in that lower eGFR has been associated with higher risk (Table 2). For both outcomes, there was a trend that the HRs for eGFR measurements incorporating cystatin C were stronger than eGFRs using creatinine alone (Table 2). For incident CVD, corresponding multivariable-adjusted HRs (95% CIs) of eGFRcr-cys(ASR), eGFRcr-cys(AS) and eGFRcys(AS) were 2.23 (1.48, 3.34), 2.26 (1.51, 3.40) and 2.09 (1.38, 3.17), and HRs (95% CIs) of eGFRcr(ASR) and eGFRcr(AS) were 1.82 (1.26, 2.63) and 1.50 (1.05, 2.14), compared group of eGFR<60 with ≥90 ml/min/1.73 m^2^. For all-cause mortality, multivariable-adjusted HRs (95% CIs) of eGFRcr-cys(ASR), eGFRcr-cys(AS) and eGFRcys(AS) were 2.68 (2.16, 3.34), 2.73 (2.20, 3.39), 3.16 (2.54, 3.94), and HRs (95% CIs) of eGFRcr(ASR) and eGFRcr(AS) were 2.02 (1.64, 2.47) and 1.53 (1.25, 1.88), compared group of eGFR<60 with ≥90 ml/min/1.73 m^2^.

**Table 2:** Association of eGFR categories with incident CVD and all-cause mortality.

eGFR categories	Incident CVD			All-cause mortality		
	Events, *n* (%)	Model 1	Model 2	Events, *n* (%)	Model 1	Model 2
		HR (95%CI)	HR (95%CI)		HR (95%CI)	HR (95%CI)
2009 eGFRcr(ASR)						
≥90	197 (7.1)	1.00	1.00	489 (15.7)	1.00	1.00
60–90	155 (10.9)	0.96 (0.76, 1.20)	0.96 (0.75, 1.22)	506 (29.9)	1.04 (0.91, 1.19)	1.06 (0.92, 1.22)
<60	59 (30.0)	2.48 (1.80, 3.43)	1.82 (1.26, 2.63)	212 (66.5)	2.26 (1.89, 2.69)	2.02 (1.64, 2.47)
2021 eGFRcr(AS)						
≥90	135 (6.8)	1.00	1.00	327 (14.8)	1.00	1.00
60–90	196 (9.3)	0.86 (0.68, 1.09)	0.88 (0.68, 1.13)	608 (24.7)	0.90 (0.78, 1.04)	0.92 (0.79, 1.07)
<60	80 (26.1)	1.98 (1.45, 2.71)	1.50 (1.05, 2.14)	272 (58.8)	1.69 (1.42, 2.02)	1.53 (1.25, 1.88)
2012 eGFRcr-cys(ASR)						
≥90	253 (7.1)	1.00	1.00	653 (16.2)	1.00	1.00
60–90	112 (16.5)	1.43 (1.12, 1.84)	1.28 (0.98, 1.66)	389 (44.1)	1.45 (1.27, 1.66)	1.38 (1.19, 1.61)
<60	46 (34.1)	3.97 (2.82, 5.57)	2.23 (1.48, 3.34)	165 (75.0)	3.65 (3.03, 4.38)	2.68 (2.16, 3.34)
2021 eGFRcr-cys(AS)						
≥90	246 (7.0)	1.00	1.00	629 (16.0)	1.00	1.00
60–90	119 (15.8)	1.40 (1.10, 1.79)	1.26 (0.97, 1.63)	410 (42.6)	1.46 (1.28, 1.67)	1.39 (1.20, 1.61)
<60	46 (33.8)	4.02 (2.86, 5.65)	2.26 (1.51, 3.40)	168 (74.7)	3.69 (3.07, 4.43)	2.73 (2.20, 3.39)
2012 eGFRcys(AS)						
≥90	283 (7.5)	1.00	1.00	719 (16.9)	1.00	1.00
60–90	87 (17.4)	1.48 (1.14, 1.92)	1.22 (0.92, 1.61)	332 (49.5)	1.78 (1.54, 2.05)	1.58 (1.35, 1.85)
<60	41 (32.0)	3.76 (2.66, 5.32)	2.09 (1.38, 3.17)	156 (77.2)	4.23 (3.52, 5.09)	3.16 (2.54, 3.94)

Model 1, adjusted for sex, age. Model 2, for incident CVD, adjusted for atherosclerotic risk factors, including age, sex, current smoking, diabetes, SBP, DBP, hypertension medications, statin medications, total cholesterol, HDL. Additionally adjusted CVD history for all-cause mortality.

### Prediction of incident CVD and all-cause mortality by eGFR measures

The C-statistics of atherosclerotic risk factors (age, sex, current smoking, SBP, DBP, diabetes, total cholesterol, HDL cholesterol, antihypertensive medication, and statin medication) for incident CVDwas 0.7791, and C-statistics of atherosclerotic risk factors and CVD history for all-cause mortality was 0.7811. There was a significant discrimination improvement in C-statistics for the predictive ability of incident CVD events and all-cause mortality after adding each eGFR measure to the basic model including atherosclerotic risk factors (Table 3).

**Table 3:** C-statistics and differences of C-statistics for the prediction of incident CVD and all-cause mortality by 5 eGFR measures.

eGFR categories	Incident CVD			All-cause mortality		
	C-statistics	Difference (95% CI)	*p* value	C-statistics	Difference (95% CI)	*p* value
Basic multivariable model	0.7791			0.7811		
+ 2009 eGFRcr(ASR)	0.7843	0.0053 (0.0009, 0.0117)	<0.001	0.7857	0.0046 (0.0023, 0.0075)	<0.001
+ 2021 eGFRcr(AS)	0.7845	0.0058 (0.0011, 0.0125)	<0.001	0.7850	0.0039 (0.0017, 0.0068)	0.003
+ 2012 eGFRcr-cys(ASR)	0.7838	0.0048 (0.0008, 0.0106)	<0.001	0.7878	0.0067 (0.0036, 0.0104)	<0.001
+ 2021 eGFRcr-cys(AS)	0.7842	0.0052 (0.0009, 0.0113)	<0.001	0.7883	0.0072 (0.0039, 0.0112)	<0.001
+ 2012 eGFRcys(AS)	0.7833	0.0044 (0.0008, 0.0098)	<0.001	0.7908	0.0097 (0.0058, 0.0141)	<0.001

For incident CVD, basic multivariable model included atherosclerotic risk factors (age, sex, smoking, diabetes, SBP, DBP, hypertension medications, statin medications, total cholesterol, HDL). For all-cause mortality, basic multivariable model included atherosclerotic risk factors and CVD history. Difference and p value compared to the basic multivariable model.

We tested the improvement of risk classification across the 7.5% 10-year risk threshold for statin therapy used in AHA/ACC guidelines (24). For incident CVD events, the addition of any eGFR measures to atherosclerotic risk factors did not improve NRI (Table 4). For mortality, race and race-free eGFRs including creatinine or both creatinine and cystatin C did not improve risk classification, but eGFRcys(AS) did (NRI: 2.19, 95%CI: 0.08, 4.65%).

**Table 4:** Net reclassification index for incident CVD and all-cause mortality for 5 eGFR measures.

eGFR categories	Incident CVD			All-cause mortality		
	NRI (95% CI)	Case NRI (95% CI)	Non-case NRI (95% CI)	NRI (95% CI)	Case NRI (95% CI)	Non-case NRI (95% CI)
Basic multivariable model						
+ 2009 eGFRcr(ASR)	2.91% (−2.25%, 5.13%)	1.63% (−3.30%, 3.87%)	1.29% (0.16%, 2.60%)	0.20% (−0.75%, 2.41%)	−0.62% (−1.70%, 1.19%)	0.82% (0.20%, 1.97%)
+ 2021 eGFRcr(AS)	−0.58% (−2.61%, 5.95%)	−1.78% (−3.51%, 4.26%)	1.20% (−0.05%, 2.69%)	0.64% (−0.50%, 2.93%)	−0.17% (−1.29%, 1.91%)	0.80% (0.12%, 1.78%)
+ 2012 eGFRcr-cys(ASR)	2.21% (−2.94%, 5.05%)	0.75% (−4.24, 3.65%)	1.46% (0.18%, 2.76%)	0.75% (−0.84%, 3.22%)	−1.02% (−2.61%, 0.84%)	1.77% (0.75%, 3.42%)
+ 2021 eGFRcr-cys(AS)	0.75% (−3.32%, 4.87%)	−0.46% (−4.45%, 3.51%)	1.22% (0.12%, 2.68%)	0.70% (−0.79%, 3.16%)	−1.02% (−2.58%, 0.92%)	1.72% (0.83%, 3.28%)
+ 2012 eGFRcys(AS)	0.74% (−2.81%, 4.24%)	−0.27% (−4.14%, 2.71%)	1.02% (0.11%, 2.45%)	2.19% (0.08%, 4.65%)	−0.55% (−2.51%, 1.32%)	2.75% (1.48%, 4.51%)

For incident CVD, basic multivariable model includesatherosclerotic risk factors (age, sex, smoking, diabetes, SBP, DBP, hypertension medications, statin medications, total cholesterol, HDL). For all-cause mortality, basic multivariable model included atherosclerotic risk factors and CVD history.

## Discussion

This study represents the largest prospective cohort analysis, comparing the predictive performance of five CKD-EPI eGFR equations, both with and without race adjustments, in predicting the incident CVD and all-cause mortality within a general African American community population. Results showed that the race-free eGFRcr and eGFRcr-cys were lower than the previous eGFRcr and eGFRcr-cys included a race correction term, respectively, and a similar trend was found among Black participants in CRIC (Chronic Renal Insufficiency Cohort) study (25). Lower eGFR was associated consistently with a higher risk of incident CVD events and all-cause mortality regardless of which race or race-free CKD-EPI equations were used. The eGFRs including cystatin C were strongly associated with incident CVD and all-cause mortality than the eGFRs using creatinine alone. A significant improvement in c-statistics was observed when all eGFR measures were added to the atherosclerotic risk factors model.

The new race-free eGFR equations represent a major advance towards eliminating racial biases. In the present study, 2021 eGFRcr(AS) had the lowest eGFR and classified more people to the group of less than 60 ml/min/1.73 m^2^ compared with the other equations. Timely care is of particular importance for CKD patients. The previous race-based eGFRcr had higher eGFR and underestimated the proportion of CKD patients in Black general population, which may lead to delayed timely care, inadequate drug dosing, and less access to dialysis and kidney transplantation. Meanwhile, the eGFRcr-cys(AS) was slightly lower than eGFRcr-cys(ASR), which also had small but potentially meaningful effects on CKD events. The comparison between eGFRcr(ASR) and eGFRcr(AS) from our study is consistent with the results from other Black population studies. In a study of 2,225 African American patients, up to one in three patients would be reclassified to a more severe CKD stage using eGFRcr(AS) instead of eGFRcr(ASR) (26). Among the 2,521 Black patients in Butt’s study, the mean eGFR reduced from 75 ± 25 to 68 ± 22 ml/min/1.73m^2^, and the proportion of <60 ml/min/1.73m^2^ increased from 29.8 to 39.1% using the eGFRcr(ASR) and eGFRcr(AS) (27). In the CREDENCE (Canagliflozin and Renal Events in Diabetes with Established Nephropathy Clinical Evaluation) trial, eGFRcr(AS) was lower than eGFRcr(ASR) (52 ± 15 vs. 56 ± 16 ml/min/1.73 m^2^) in 223 Black participants (28). However, different comparison results were observed in other studies. eGFRcr(AS) is higher than eGFRcr(ASR) in Thai patients (29). eGFRcr(AS) has a higher estimation of GFR than eGFRcr(ASR) among Asian cohorts and the European population (30–32). A study indicates that among these five eGFR measures, eGFRcys(AR) has the lowest estimate in the Chinese population (33).

We have demonstrated that eGFR is independently associated with incident CVD and all-cause mortality, regardless of the equations. The adjusted shape revealed that the relationship between eGFR measures and incident CVD was monotonic. Concerning mortality, the shape exhibited an initially negative association and followed by a flat trend, except for eGFRcr(ASR) and eGFRcr(AS), which showed increased mortality after reaching 105 ml/min/1.73m^2^. Other studies showed a similar trend. A U-shaped association was found between eGFRcr(ASR) and mortality, while a monotonic association in eGFRcys(AS) and eGFRcr-cys(ASR) (34, 35). For CVD, an inflection point at approximately 90 ml/min/1.73m^2^ was found for eGFRcr(AS), below and above which there were increasing hazards (36). Compared with normal eGFR, high eGFRcys was associated with a lower risk of CVD (37), and eGFRcr(ASR) ≥105 ml/min/1.73m^2^ was associated with a 2-fold increased mortality (38). In the meantime, a negative linear relationship between eGFR measures and CVD events and mortality was observed in other studies (15, 39).

Our analysis showed that significant renal dysfunction (any eGFR<60 ml/min/1.73 m^2^) is associated with incident CVD events, supporting the published results from other studies (13, 14). However, mild renal dysfunction (60–90 ml/min/1.73 m^2^) did not prove to be an independent risk factor for incident CVD in our study, which is similar to other studies. The prospective Reykjavik study did not show an association between eGFR of 60–90 ml/min/1.73 m^2^ and risk of coronary heart disease (40). A large-scale retrospective study based on 10,909 subjects with normal to mildly reduced renal function found that the association between lower eGFR and new onset of CVD is no longer significant after adjusting other cardiovascular risk factors (41). Whereas, a different result was reported in ARIC (Atherosclerosis Risk in Communities) study (42), FHS (The Framingham Heart Study) (43), suggesting that a lower eGFR even in the normal or mildly impaired range is associated with a higher incidence of CVD. These controversial results may be related to dissimilar populations.

Keeping with other published data from large cohort studies (11, 12, 44–46), we have shown that lower eGFR was associated with a high risk of all-cause mortality. Even milder impairments in renal function (60–90 ml/min/1.73 m^2^) based on eGFRcr-cys(ASR) or eGFRcr-cys(AS) and eGFRcys(AS) have also been shown to increase the risk of all-cause mortality, while eGFRcr(ASR) and eGFRcr(AS) are not. Controversial findings have been reported in other studies. In the AusDiab (Austrialian Diabetes, Obesity and Lifestyle) study, eGFR of 60–90 ml/min/1.73 m^2^ using eGFRcr(ASR), eGFRcys(AS) and eGFRcr-cys(ASR) was not associated with all-cause mortality (47). In older adults of Good Aging in Skåne study, no significant association was observed between moderate renal dysfunction and mortality [adjusted HR (95% CI): 0.90 (0.67–1.21) for eGFRcr-cys(ASR) (>90 vs. 60–89 ml/min/1.73 m^2^)] (46).

The eGFRs based on both creatinine and cystatin or cystatin C alone had a higher association with risks for incident CVD and all-cause mortality than GFR estimates using creatinine alone. Compared group of eGFR<60 with ≥90 ml/min/1.73m^2^, multivariable-adjusted HRs of eGFRcr-cys(ASR), eGFRcr-cys(AS) and eGFRcys(AS) were mostly 2.09 or higher, HRs of eGFRcr(ASR) and eGFRcr(AS) were 1.82 and 1.50 for incident CVD, and HRs of eGFRcr-cys(ASR), eGFRcr-cys(AS) and eGFRcys(AS) were 2.68 to 3.16, and eGFRcr(ASR) and eGFRcr(AS) were 2.02 and 1.53 for all-cause mortality. This occurred in studies reported by Shlipak et al. (39) and Barr et al. (47), which compared eGFRcr(ASR), eGFRcr-cys(ASR), eGFRcys(AS). A significant improvement in C-statistics when any eGFR measures were added to a base risk factors model in this study. Only eGFRcys(AS) improved the net reclassification of all-cause mortality in our study. Previous studies have shown a similar result that eGFRCys(AS) has more predictive utility for all-cause mortality than eGFRCr(ASR) and eGFRcr-cys(ASR) (15, 36, 47, 48). In the meantime, cystatin C is considered a more sensitive marker of kidney function and is less influenced by muscle mass, age, gender and protein diet compared with creatinine (49, 50). eGFRcys(AS) indicated a more significant association with ischaemic stroke compared to eGFRCr(ASR). This effect was more pronounced in women than men. The measurement of cystatin C may enhance risk stratification for ischaemic stroke and improve clinical treatment in a general population, especially for women (51). NKF and ASN recommended nationwide efforts to widely measure and use cystatin C, especially for adults at risk for or have CKD (9). Therefore, the measure of cystatin C may be necessary and cystatin C-based eGFRcys might be more appropriate for predicting CVD and mortality in Black population.

## Strengths and limitations

Our study has strengths and limitations. The JHS is a large community-based sample of African Americans, with a high prevalence of CKD and CVD. The new CKD-EPI eGFR without race was developed to address racial bias. Therefore, JHS is one of the best populations to evaluate the performance of the new eGFRs in African Americans. Furthermore, serum creatinine and cystatin C were collected at baseline and GFR can be evaluated with five CKD-EPI eGFRs in the JHS. Some limitations need to be mentioned. First, there was no available data on gold standard measured GFR, which limited our ability to validate the race and race-free eGFRs against measured GFR and make inferences on which one was most appropriate for our study. In addition, our results were obtained from an African American cohort in Mississippi and may not be generalizable to Black individuals from other communities or countries.

## Conclusion

In summary, Reducing eGFR was related to a higher incidence of CVD events and mortality, and e GFRcr-cys(ASR), eGFRcr-cys(AS) and eGFRcys(AS) strengthened the association. Cystatin C-based eGFR equations might be more appropriate for predicting CVD and mortality in Black population.

## Data Availability

The datasets presented in this article are not readily available because the data are from the JHS. The JHS data are available to researchers with approved manuscript proposals. Requests to access the datasets should be directed to the JHS Committee at https://www.jacksonheartstudy.org/Research/Study-Data/Data-Access.

## Glossary

AS: Age and sex
ASR: Age, sex and race
BMI: Body mass index
CI: Confidence interval
CKD: Chronic kidney disease
cr: Creatinine
CVD: Cardiovascular disease
cys: Cystatin C
DBP: Diastolic blood pressure
eGFR: Estimated glomerular filtration rate
GED: General education development
HDL: High-density lipoproteins
HR: Hazard ratio
hsCRP: High-sensitivity C-reactive protein
IQR: Interquartile range
LDL: Low-density lipoproteins
SBP: Systolic blood pressure
SD: Standard deviation
