# Supplementary material for: Prediction of cardiovascular events and all-cause mortality using race and race-free estimated glomerular filtration rate in African Americans: the Jackson Heart Study
Source: Front Med (Lausanne). 2024 Oct 31;11:1432965. doi: 10.3389/fmed.2024.1432965 (PMC11560791; doi:10.3389/fmed.2024.1432965)
Supplement: Supplementary file 1 [file Table_1.DOCX]

Table S1. CKD-EPI equations for estimating GFR

| **Equations** | **Equation** |
| --- | --- |
| eGFRcr(ASR) | 141 × min(Scr/κ, 1)^α^ × max(Scr/κ, 1)^-1.209^ × 0.9929^Age^ × 1.018 [if female] × 1.159 [if Black]  Scr is serum creatinine, κ is 0.7 for females and 0.9 for males, α is -0.329 for females and -0.411 for males, min indicates the minimum of Scr/κ or 1, and max indicates the maximum of Scr/κ or 1. |
| eGFRcr(AS) | GFR = 142 × min(Scr/κ, 1)^α^ × max(Scr/κ, 1)^-1.200^ × 0.9938^age^ × 1.012 [if female]  Scr is serum creatinine, κ is 0.7 for females and 0.9 for males, α is -0.241 for females and  -0.302 for males, min indicates the minimum of Scr/κ or 1, max indicates the maximum of Scr/κ or 1. |
| eGFRcr-cys(ASR) | 135 × min(Scr/κ, 1)^α^ × max(Scr/κ, 1)^−0.601^ × min(Scys/0.8, 1)^−0.375^ × max(Scys/0.8, 1)^−0.711^ × 0.9952^Age^× 0.969 [if female] × 1.08 [if Black]  Scr is serum creatinine, Scys is serum cystatin C, κ is 0.7 for females and 0.9 for males, α is  -0.248 for females and -0.207 for males, min indicates the minimum of Scr/κ or 1, and max indicates the maximum of Scr/κ or 1, min(Scys/0.8, 1) indicates the minimum of Scys/0.8 or 1, max(Scys/0.8, 1) indicates the maximum of Scys/0.8 or 1. |
| eGFRcr-cys(AS) | 135 × min(Scr/κ, 1)^α^ × max(Scr/κ, 1)^−0.544^ × min(Scys/0.8, 1)^−0.323^ × max(Scys/0.8, 1)^−0.778^ × 0.9961^Age^× 0.963 [if female]  Scr is serum creatinine, Scys is serum cystatin C, κ is 0.7 for females and 0.9 for males, α is  -0.219 for females and -0.144 for males, min indicates the minimum of Scr/κ or 1, and max indicates the maximum of Scr/κ or 1, min(Scys/0.8, 1) indicates the minimum of Scys/0.8 or 1, max(Scys/0.8, 1) indicates the maximum of Scys/0.8 or 1. |
| eGFRcys(AS) | 133 × min(Scys/0.8, 1)^−0.499^ × max (Scys/0.8, 1)^−1.328^ × 0.9962^Age^ × 0.932 [if female]  Scys is serum cystatin C, min indicates the minimum of Scys/κ or 1, max meansmaximum of Scys/κ or 1. |

Serum creatinine is expressed in mg/dL, Serum cystatin C is expressed in mg/L.
